# Association between atherogenic index of plasma and all-cause mortality and specific-mortality: a nationwide population‑based cohort study

**DOI:** 10.1186/s12933-024-02370-4

**Published:** 2024-07-27

**Authors:** Fang-Fei You, Jian Gao, Yi-Ning Gao, Zhi-Hao Li, Dong Shen, Wen-Fang Zhong, Jin Yang, Xiao-Meng Wang, Wei-Qi Song, Hao Yan, Hao-Yu Yan, Jia-Hao Xie, Huan Chen, Chen Mao

**Affiliations:** 1 Department of Epidemiology, School of Public Health, Southern Medical University, 1838 Guangzhou North Road, Guangzhou, 510515 Guangdong China; 2 Department of Laboratory Medicine, Microbiome Medicine Center, Zhujiang Hospital, Southern Medical University, Guangzhou, Guangdong China

**Keywords:** Atherogenic index of plasma; all-cause mortality, Specific mortality, NHANES

## Abstract

**Background:**

Atherogenic index of plasma (AIP), a marker of atherosclerosis and cardiovascular disease (CVD). However, few studies have investigated association between AIP and all-cause mortality and specific-mortality in the general population.

**Methods:**

This study included data from 14,063 American adults. The exposure variable was the AIP, which was defined as log10 (triglycerides/high-density lipoprotein cholesterol). The outcome variables included all-cause mortality and specific-mortality. Survey-weighted cox regressions were performed to evaluate the relation between AIP and all-cause mortality and specific-mortality. Weighted restricted cubic spline was conducted to examin the non-linear relationship.

**Results:**

During 10 years of follow-up, we documented 2,077, 262, 854, and 476 cases of all-cause mortality, diabetes mortality, CVD mortality and cancer mortality, respectively. After adjustment for potential confounders, we found that atherogenic index of plasma (AIP) was significantly associated with an increased risk of diabetes mortality when comparing the highest to the lowest quantile of AIP in female (*p* for trend = 0.001) or participants older than 65 years (*p* for trend = 0.002). AIP was not significantly associated with all-cause mortality, CVD mortality and cancer mortality (*p* > 0.05). Moreover, a non-linear association was observed between AIP and all-cause mortality in a U-shape (*p* for non-linear = 0.0011), while a linear relationship was observed with diabetes mortality and non-diabetes mortality (*p* for linear < 0.0001).

**Conclusions:**

In this study, there is a no significant association between high AIP levels and a high risk of all-cause and cardiovascular mortality. Besides, a higher AIP was significantly associated with an increased risk of diabetes mortality, which only found in women older than 65 years. AIP was associated with all-cause mortality in a U-shape. This association could be explained by the finding that higher AIP predicted a higher risk of death from diabetes, and that lower AIP predicted a higher risk of death from non-diabetes causes.

**Supplementary Information:**

The online version contains supplementary material available at 10.1186/s12933-024-02370-4.

## Highlight

- We used a large national database and a prospective cohort study with a long follow-up period.
- Higher AIP was significantly associated with an increased risk of diabetes mortality, only in women older than 65 years.
- There is a no significant association between high AIP levels and a high risk of all-cause and cardiovascular mortality.
- AIP was associated with all-cause mortality in a U-shape.
- This finding suggest that controlling AIP levels may have a positive effect on reducing diabetes mortality.

**Supplementary Information:**

The online version contains supplementary material available at 10.1186/s12933-024-02370-4.

## Introduction

Dyslipidemia is one of the important modifiable risk factors for Myocardial infarction (MI) [1], and is associated with the risk of hypertension [2] and diabetes [3]. The typical features of dyslipidemia include elevated levels of total cholesterol (TC), triglyceride (TG), low-density lipoprotein cholesterol (LDL-C), as well as a decreased concentration of high-density lipoprotein cholesterol (HDL-C) [4, 5]. Atherogenic index of plasma (AIP) was first proposed by Dobiásová and Frohlich [6], it is an independent predictive marker for rapid plaque progression [7], and was positively related to the risk and severity of coronary atherosclerotic disease [8]. AIP is calculated as log10 (TG/HDL), it not only reflects levels of TG and HDL-C, but also serves as a powerful predictor of dyslipidemia [9].

In recent years, numerous studies have shown that the AIP is a powerful biomarker for predicting CVD diseases [10–13], diabetes [14, 15] and metabolic syndrome [16]. It was suggested that AIP could predict the size of lipoprotein particles [17], showing a positive correlation with the risk of MI [18, 19]. Higher AIP is significantly and positively associated with the risk of prehypertension in a Japan population [20]. A meta-analysis revealed that patients with type 2 diabetes mellitus (T2DM) had significantly higher AIP values compared to those without T2DM [21]. AIP was also proved positively correlated with arterial stiffness in patients with hypertension [22]. In addition, AIP is elevated in patients with obstructive sleep apnoea and is related to disease severity [23]. A study has explored the association between AIP and all-cause mortality and CVD mortality in patients with hypertension [24]. However, limited studies have characterized the exposures to AIP and their implications for all-cause and specific mortality in general population. Besides, the nonlinear relationship between AIP and mortality needs to be explored.

Herein, this study aims to explore the relationship and nonlinear associations between AIP and all-cause mortality and specific mortality, and further assess these relationships in subgroups of age and sex using a large-scale population dataset from the National Health and Nutrition Examination Survey (NHANES).

## Methods

### Study Population

Data used in this retrospective cohort study were all from the NHANES database [25]. NHANES is a national research program conducted by the National Center for Health Statistics (NCHS), it selects a group of representative American people by a multistage, stratified, subgroup probability sampling, and aims to assess the health and nutrition status of adults and children in the United States [26]. This cohort study enrolled participants aged 18 years during the 8 cycles of NHANES 2003–2018. The individuals with missing sociodemographic characteristics, missing TG and HDL-C measurements used to calculate AIP, and with no linked mortality data were excluded from the analysis. 14,063 participants including 7075 women and 6988 men were included in this study. The NHANES protocol was revised and approved by the Ethics Review Committee of the NCHS, and all participants provided written informed consent [27]. More details of the study can be accessed online: www.cdc.gov/nchs/nhanes/irba98.htm.

### Definitions of the exposure and outcome variables

The exposure variable was the AIP, which was mathematically derived from lg[TG(mmol/L)/HDL-C(mmol/L)] with both TG and HDL-C levels are expressed in mmol/L. [28]. According to a standardized protocol from the Centers for Disease Control and Prevention (CDC), serum HDL-C was measured by direct immunoassay or precipitation [29]. Fasting venous blood was drawn from each subject for TG measurement.Subsequently, we classified the study population into four groups according to the AIP quartiles.

The study outcomes were all-cause mortality, diabetes mortality, CVD mortality, and cancer mortality. The information of mortality in the NHANES are available from the National Death Index (NDI) death certificate records (www.cdc.gov/nchs/data-linkage/mortality-public.htm). The corresponding mortality information for each participant was identified through linkage to the National Mortality Index up to 31 December 2019. The International Classification of Diseases (ICD)-10 was used to determine disease-specific deaths. Cardiovascular disease mortality was defined as any death related to heart disease, cerebrovascular disease, and/or hypertension. Death from heart disease was defined as codes I00-09, I11, I13 and I20-51, and death from cerebrovascular disease was defined ascodes I60- I69 according to ICD-10. Diabetes mortality was defined as codes E10-E14, and cancer mortality was defined as codes C00-C97.

### Potential covariates

Sociodemographic characteristics included age (years; continuous), sex (men/women), race/ethnics (Mexican American, non-Hispanic white, non-Hispanic black and others), education level (less than 9th grade, 9-11th grade, high school, college, and college graduate or above) and annual household income (under $20,000, $20,000 to $45,000, $45,000 to $75,000, $75,000 to $100,000, and over $100,000). The health behaviors included current smoking(no/yes), alcohol drinking(no/yes), and moderate to vigorous activity regularly (no/yes). Other potential confounders were body mass index (kg/m^2^; continuous), self-reported CVD (no/yes), diabetes(no/yes), hypertension(no/yes), and high cholesterol (no/yes).

### Statistical analysis

All analyses were performed with the incorporation of sample weights, stratification, and cluster to account for the complex survey design. Continuous variables were presented as weighted mean (± SE), and categorical variables were presented as unweighted frequency, weighted frequency of participants (weighted percentage). Participants were categorized by AIP quartiles. To evaluate the relation between AIP and risk of outcomes, survey-weighted Cox regressions were performed. Model 1 represented the unadjusted data. In Model 2, the data were adjusted for age and sex. In Model 3, the results were adjusted for age, sex, race/ethnicity, education level, annual household income, body mass index, current smoking, current alcohol drinking, moderate to vigorous activity regularly, self-reported of CVD, diabetes, hypertension, and high cholesterol. The results from the Cox regression analysis are presented as hazard ratios (HRs)and 95%confidence intervals (CIs). We used a weighted restricted cubic spline to explore the potential dose-response pattern, selecting 3 knots (10th, 50th, and 90th percentiles of AIP) to smooth the curve. If the relationship was nonlinear, a threshold effect analysis is performed, which implies that we utilized two-piecewise Cox proportional risk model on both sides of the infection point to investigate the association between AIP and the risk of all-cause mortality and specific mortality.

We performed a stratified analysis to estimate potential modification effects according to sex (male or female) and age (< 65 or ≥ 65 years). Several sensitivity analyses were conducted. First, in order to minimise the influence of reverse causation, we conducted sensitivity analysis by excluding participants who died during the first two years of follow-up. Second, we evaluated the association between AIP and all-cause mortality and specific-mortality excluding participants self-reported CVD at baseline. Third, we further evaluated the association between AIP and all-cause mortality and specific-mortality with an weighting procedure for the morning fasting subgroup. Fourth, we examined the association between AIP and all-cause mortality and specific-mortality additionally adjusted for self-reported cancer status. All statistical analyses were conducted using R version 4.2.0 (R Foundation for Statistical Computing), with a 2-tailed alpha value of 0.05 considered statistically significant.

## Results

### Baseline characteristics

During 10 years of median follow-up, we documented 2,077, 262, 854, and 476 cases of all-cause mortality, diabetes mortality, CVD mortality and cancer mortality, respectively. The baseline characteristics of participants stratified by gender are shown in Table 1. Compared with the male participants, the female participants were more likely older, less current smokers or drinkers, more people with higher education levels and less household income. These individuals were also more likely to have higher levels of BMI, exercise regularly, and have less prevalence of ever had CVD, diabetes, hypertension, and high cholesterol. Besides, the baseline characteristics of participants further divided by age/ AIP quintile categories are shown in Tables 1S, 2S and 3S (Supplementary Files).

**Table 1:** Baseline characteristics of participants stratified by gender

	Total	Male	Female
**No. of participants**	14,063	6988	7075
**Age (mean (SE))**	46.64 (0.29)	45.77 (0.33)	47.48 (0.31)
**Race/Ethnics (%)**			
Mexican American	2440, 45706447.53 (8.4%)	1223, 24354974.09 (9.1%)	1217, 21351473.44 (7.7%)
Non-Hispanic white	6586, 383896661.6 (70.4%)	3328, 189192920.7 (70.9%)	3258, 194703740.9 (70.0%)
Non-Hispanic black	2815, 55847231.01 (10.2%)	1371, 24785488.16 (9.3%)	1444, 31061742.84 (11.2%)
**Current smoking (%)**	3296, 133071265.6 (24.4%)	2044, 80728576.04 (30.2%)	1252, 52342689.59 (18.8%)
**Current drinking (%)**	8989, 384959429.1 (70.6%)	5232, 213710414.9 (80.0%)	3757, 171249014.2 (61.6%)
**Education level (%)**			
Less than 9th grade	1519, 31864201.03 (5.8%)	773, 161120448.60 (6.0%)	746, 15752152.43 (5.7%)
9-11th grade	2097, 62901121.57 (11.5%)	1086, 32134256.58 (12.0%)	1011, 30766864.99 (11.1%)
High school	3556, 131005326.2 (24.0%)	1858, 66916082.23 (25.1%)	1698, 64089243.93 (23.0%)
College	3952, 168543830.8 (30.9%)	1806, 78111485.68 (29.3%)	2146, 90432345.10 (32.5%)
College graduate or above	2925, 150538469.6 (27.6%)	1459, 73591566.12 (27.6%)	1466, 76946903.49 (27.7%)
**Annual household income (%)**			
Under $20,000	3031, 77881907.08 (14.3%)	1309, 31454473.96 (11.8%)	1722, 46427433.12 (16.7%)
$20,000 to $45,000	4644, 154610812.9 (28.4%)	2326, 73260388.52 (27.4%)	2318, 81350424.37 (29.2%)
$45,000 to $75,000	2611, 119775139.9 (22.0%)	1338, 60175124.83 (22.5%)	1273, 59600015.11 (21.4%)
$75,000 to $100,000	1718, 94662986.08 (17.4%)	917, 49713299.60 (18.6%)	801, 44949686.47 (16.2%)
Over $100,000	1440, 80542142.00 (14.8%)	788, 43816570.04 (16.4%)	652, 36725571.96 (13.2%)
**BMI (mean (SE))**	29.27(0.11)	29.11(0.15)	29.43(0.14)
**Exercised regularly (%)**	6713, 231234956.0 (42.4%)	3149, 107298490.4 (40.2%)	3564, 123936465.7 (44.6%)
**Ever had CVD (%)**	1501, 47991950.19 (8.8%)	855, 26949603.08 (10.1%)	646, 21042347.11 (7.6%)
**Ever had diabetes (%)**	2278, 67279390.88 (12.3%)	1206, 35323992.08 (13.2%)	1072, 31955398.80 (11.5%)
**Ever had hypertension (%)**	5676, 99897161.30 (36.7%)	2813, 99219152.42 (37.2%)	2863, 100678008.9 (36.2%)
**Ever had high cholesterol (%)**	6720,261360137.80 (47.9%)	3732, 144683718.9 (54.2%)	2988, 116676418.9 (42.0%)
**AIP (mean (SE))**	– 0.03(0.01)	0.04(0.01)	– 0.10(0.01)

Continuous variables were presented as weighted mean (± SE), and categorical variables were presented as unweighted frequency, weighted frequency of participants (weighted percentage)

### Association between atherogenic index of plasma and all-cause mortality and specific mortality

After adjustment for potential confounders, the associations of AIP and all-cause mortality and specific mortality stratified by gender or age are presented in Table 2.

**Table 2:** Association between AIP and all-cause mortality and specific-mortality stratified by gender or age

	Male (*N* = 6988)		Female (*N* = 7075)		< 65 year-old (*N* = 10,978)		≥ 65 year-old (*N* = 3085)	
	Events/total	HR (95%CI)^a^	Events/total	HR (95%CI)^a^	Events/total	HR (95%CI)^a^	Events/total	HR (95%CI)^a^
**All-cause mortality**								
Quartile 1	181/1275	Reference	192/2230	Reference	99/2882	Reference	274/623	Reference
Quartile 2	287/1692	1.01 (0.79,1.29)	221/1860	0.78 (0.60,1.04)	137/2769	0.87 (0.60,1.27)	371/783	0.92 (0.75,1.12)
Quartile 3	324/1808	0.91 (0.73,1.14)	259/1700	0.94 (0.71,1.24)	158/2610	0.94 (0.62,1.41)	425/898	0.92 (0.75,1.12)
Quartile 4	402/2213	1.09 (0.87,1.36)	211/1285	0.92 (0.71,1.21)	222/2717	1.06 (0.74,1.51)	391/781	0.98 (0.82,1.16)
*p* for trend^b^		0.988		0.384		0.351		0.782
**Diabetes mortality**								
Quartile 1	12/1275	Reference	15/2230	Reference	10/2882	Reference	17/623	Reference
Quartile 2	19/1692	0.93 (0.38,2.27)	22/1860	1.22 (0.52,2.84)	15/2769	0.87 (0.30,2.46)	26/783	0.87 (0.45,1.67)
Quartile 3	38/1808	1.14 (0.54,2.44)	27/1700	1.28 (0.60,2.73)	22/2610	0.73 (0.24,2.22)	43/898	1.37 (0.77,2.44)
Quartile 4	86/2213	1.78 (0.79,4.04)	43/1285	2.86 (1.38,5.94)**	63/2717	1.70 (0.61,4.77)	66/781	2.25 (1.19,4.24)*
*p* for trend^b^		0.118		0.001		0.066		0.002
**CVD mortality**								
Quartile 1	69/1275	Reference	79/2230	Reference	26/2882	Reference	122/623	Reference
Quartile 2	121/1692	1.23 (0.83,1.84)	79/1860	0.75 (0.54,1.05)	44/2769	1.13 (0.57,2.24)	156/783	0.90 (0.68,1.20)
Quartile 3	145/1808	0.98 (0.70,1.37)	103/1700	0.91 (0.64,1.30)	56/2610	1.11 (0.55,2.24)	192/898	0.97 (0.73,1.29)
Quartile 4	160/2213	1.10 (0.75,1.61)	98/1285	1.00 (0.70,1.42)	81/2717	1.26 (0.65,2.46)	177/781	1.03 (0.79,1.33)
*p* for trend^b^		0.675		0.124		0.410		0.518
**Cancer mortality**								
Quartile 1	38/1275	Reference	42/2230	Reference	20/2882	Reference	60/623	Reference
Quartile 2	75/1692	1.10 (0.71,1.71)	51/1860	0.74 (0.39,1.40)	49/2769	2.15 (0.94,4.95)	77/783	0.87 (0.57,1.34)
Quartile 3	72/1808	1.02 (0.64,1.65)	59/1700	0.91 (0.49,1.72)	41/2610	1.81 (0.76,4.32)	90/898	0.83 (0.54,1.28)
Quartile 4	96/2213	1.42 (0.87,2.32)	43/1285	1.01 (0.55,1.85)	54/2717	2.61 (1.09,6.28)*	85/781	0.92 (0.62,1.38)
*p* for trend^b^		0.093		0.530		0.037		0.965

* *p* < 0.05

** *p* < 0.01

^a^ Adjusted for age, sex, race/ethnicity, education level, annual household income, body mass index, current smoking, current alcohol drinking, moderate to vigorous activity regularly, self-reported of CVD, diabetes, and high cholesterol

^b^ Test for trend based on variable containing median value for each quintile

CI, confidence interval; HR, hazard ratios

In the sex-specific analyses **(**Table 2**)**, there is an association between higher quartiles of AIP and diabetes mortality in female. Compared with the first quartile group, the risk of diabetes mortality was significantly increased in higher quartile groups, with the HRs and 95%CIs were 1.22(0.52,2.84), 1.28(0.60,2.73), and 2.86(1.38,5.94) respectively in Q2, Q3, and Q4 group (*p* for trend = 0.001). However, the AIP of male participants was not related to all the other outcomes (*p* > 0.05).

In addition, in the age-specific analyses, compared to the reference group, the participants in Q3 who were older than 65 years had a HR of 1.37(0.77,2.44), while those in Q4 had a significantly higher risk of diabetes mortality, with a HR of 2.25(1.19,4.24). However, the relationship was not found in < 65 year-old group. In fully adjusted model, there is no correlation between AIP and all-cause or CVD mortality. For participants with Q4, the HRs (95% CI) were 0.98(0.82,1.16) for all-cause mortality, 1.03(0.79,1.33) for CVD mortality in ≥ 65 year-old group.

### Nonlinear associations between atherogenic index of plasma and outcomes

**Fig. 1:**
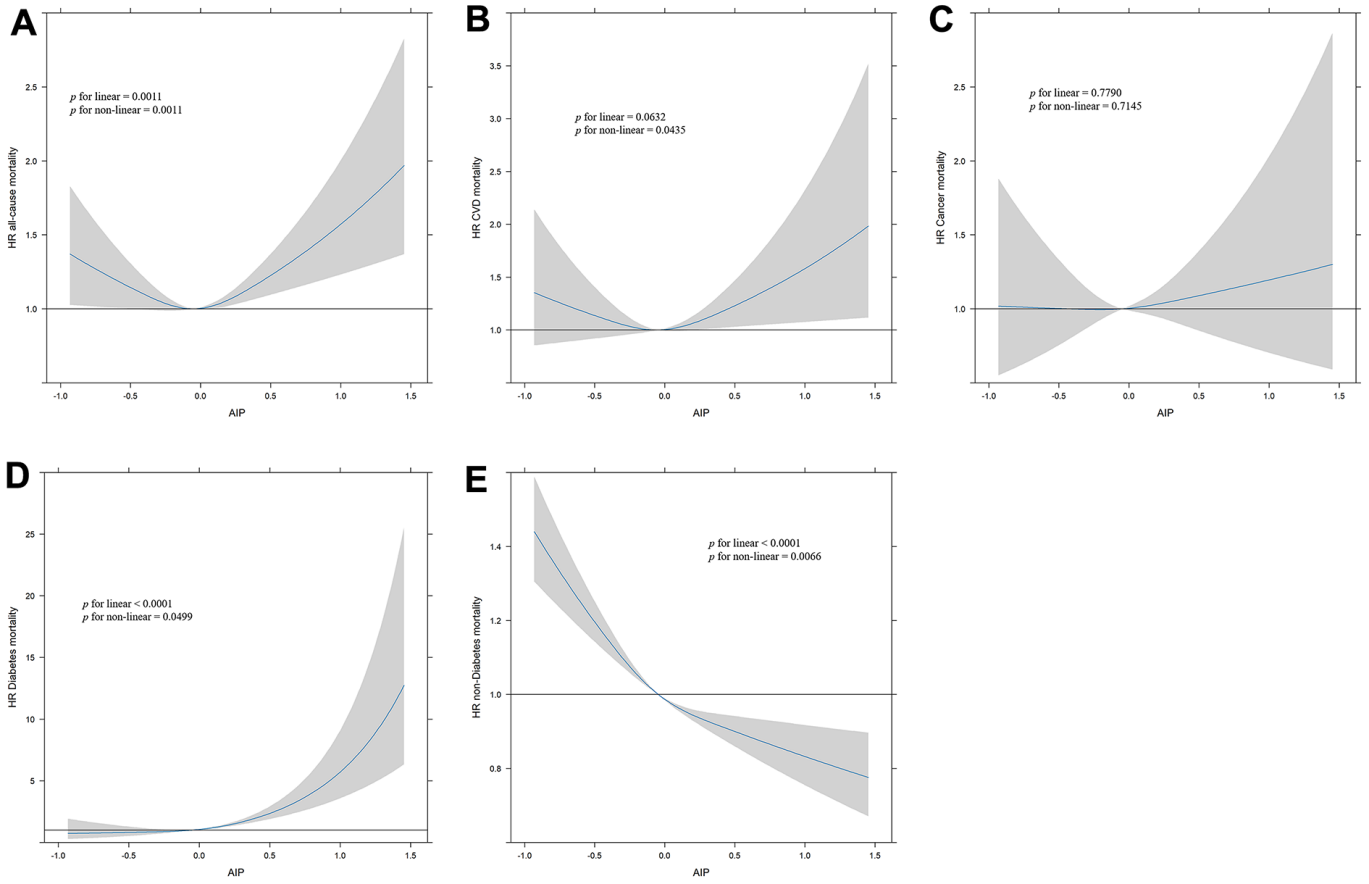
Dose-response curves of AIP and all-cause mortality and specific-mortality. A restricted cubic spline was fitted to model each curve, with 3 knots fixed at the 10th, 50th, and 90th percentiles for all smooth curves. Solid lines represent the point estimates of HRs for incident all-cause mortality (**A**), CVD mortality (**B**), cancer mortality (**C**), diabetes mortality (**D**), and non-diabetes mortality (**E**), while shadows represent corresponding 95% CIs. *p* values were calculated using the Anova test

To better explain the observed nonlinear association, we further analyzed the atherogenic index of plasma as a continuous variable using weighted cubic spline regression adjusting for all covariates mentioned in the "Methods" section. As shown in Fig. 1, there are significant nonlinear dose-response patterns between AIP and all-cause mortality, and CVD mortality (*p* for non-linear = 0.0011 and 0.0435), however, no non-linear dose-response pattern was observed between AIP and cancer mortality (*p* for non-linear = 0.7145). AIP showed a linear relationship with the risk of diabetes mortality, meaning that as AIP increased, the risk of diabetes mortality increased (*p* for linear < 0.0001). Furthermore, a non-linear and L-type association was detected between AIP and non-diabetes mortality and decreased AIP was significantly associated with increased risk of non-diabetes mortality. Building upon these findings, we performed a threshold effect analysis to further validate this U-shaped nonlinear association between AIP and mortality. Our results delineated specific thresholds: the lowest AIP associated with increased risks of all-cause mortality and CVD mortality were identified as -0.057 **(**Table 3**)**.

**Table 3:** Threshold effect analysis of AIP on all-cause and CVD mortality

	HR (95%CI)^ab^	*p*
Fitting by the two-piecewise Cox proportional risk model		
Inflection point	– 0.057	
**AIP <– 0.057 (N = 6871)**		
All-cause mortality	0.93 (0.85,1.02)	0.127
CVD mortality	0.93 (0.80,1.08)	0.341
**AIP ≥– 0.057 (N = 7192)**		
All-cause mortality	1.08 (1.01,1.16)	0.029
CVD mortality	1.09 (0.98,1.22)	0.123

^a^ Estimated as the beta coefficient for standardized AIP using the z-score

^b^ Adjusted for age, sex, race/ethnicity, education level, annual household income, body mass index, current smoking, current alcohol drinking, moderate to vigorous activity regularly, self-reported of CVD, diabetes, hypertension, and high cholesterol

### Sensitivity analyses

Sensitivity analyses showed no substantial change after adjusting for covariables in Model 3 **(Tables 4S, 5S, 6S and 7S)**. After excluding participants who died during the first two years of follow-up, participants in Q4 have a much higher risk of diabetes mortality compared with the lowest quartile in all models, with HRs of 6.35(3.14,12.84), 4.54(2.21,9.35), and 2.96(1.45,6.08) in Model 1, 2, and 3. The results remained statistically significant with a weighting procedure for the morning fasting subgroup, the HRs were 4.76(2.57,8.82), 3.33(1.79,6.21) and 2.07(1.09,3.94) respectively in the highest quartile. This positive association was still observed in all models after excluding participants with CVD diagnosed at baseline or additionally adjusted for self-reported cancer status.

## Discussion

To our knowledge, this is the first study to investigate the relationships of atherogenic index of plasma with all-cause mortality and specific-mortality in the general population. In this prospective cohort study of 14,063 individuals from the NHANES study, we found that higher AIP was associated with an increased risk of diabetes mortality. Compared with the first quartile group, the risk of diabetes mortality was significantly increased in higher quartile groups. However, the association was only found in women older than 65 years. After adjusting for potential confounders, we found a U-shaped association between AIP and all-cause mortality. This association could be explained by the finding that higher AIP predicted a higher risk of death from diabetes mortality, and that lower AIP predicted a higher risk of death from non-diabetes mortality.

AIP, the ratio between TG to HDL-C on a logarithmic scale, quantifies one’s ability to metabolize glucose and lipid [30]. It is reported elevated AIP is associated with higher risk of carotid atherosclerosis in community-based population [31]. A meta-analysis showed that AIP was a more accurate predictor of diabetes risk compared to other lipid components [21]. Fu et al. confirmed that patients with T2DM are more likely to have cardiovascular risk factors, such as hyperlipidemia, and therefore could be used as an reliable predictor for the prognosis of T2DM patients in long-term follow-up [32]. Across-sectional study demonstrated a J-shaped association between AIP and T2DM, higher AIP was significantly associated with a higher risk of T2D in patients with − 0.47 < AIP < 0.45 [33]. Our study found that AIP was significantly associated with diabetes mortality and increased with higher AIP. Our results are consistent with the findings of another NHANES study, the risk of prediabetes and diabetes increased gradually with the increase in the AIP [14].

The subgroup analysis suggests that the positive association between AIP and diabetes mortality only exists among women older than 65 years. This is consistent with the findings of an international study from 193 countries, which also showed higher T2DM-related mortality in women [34]. Possible mechanisms include physiologic differences between men and women and estrogenic changes. When TG/HDL-C is used as a continuous variable, women have a lower threshold for developing diabetes [35] and a stronger correlation [36]. Women also have a more severe diabetes-related vascular risk [37]. They may experience prolonged metabolic dysfunction prior to the diagnosis of type 2 diabetes, which may lead to a higher risk of diabetes-related vascular complications in women [38]. Compared to men, women have a higher risk of developing end-stage renal disease associated with diabetes [39]. Another possible reason for this is that gender differences in the management and treatment of diabetes tend to be unfavorable to women [40]. Estrogens can increase hepatic insulin sensitivity, increase insulin release, and prevent β-cell apoptosis [41]. The postmenopausal decline in estrogen levels in older women can lead to disturbances in glucose and lipid metabolism and an increased risk of diabetes [33]. Although estrogen levels are lower than in women, local concentrations may be much higher at the site of production and/or action [42]. This may explain why no association between AIP and diabetes has been observed in men over 65 years of age. The results we observed need to be explored in further animal experiments and population trials.

It is reported AIP is an independent predictor of CVD events [11] and mortality [28], the underlying mechanism can be explained by the correlation of this index with lipoprotein particle size: it is negatively correlated with LDL cholesterol particle diameter [43, 44]. In a research of middle-aged and elderly individuals from Lithuania, it was shown that the risk of CVD mortality significantly increased in males with the highest AIP quintile compared to those in the lowest quintile [45]. However, we did not find that AIP was associated with CVD mortality in our study. Similarly, a research among Koreans found that after controlling for traditional risk variables, the correlation between AIP and CVD mortality became insignificant, even though there was an increase in HR with greater AIP [46]. This may be due to a relatively short follow-up period, it may not be sufficient to comprehensively examine the relationship between AIP and CVD mortality, and longer follow-up may yield different results.

In addition, we found a U-shaped relationship between AIP and all-cause mortality and CVD-specific mortality. In a retrospective cohort study, both low and high levels of AIP were associated with increased risks of all-cause mortality and CVD mortality in patients with hypertension, which is consistent with our findings [24]. Due to AIP is calculated by serum TG and HDL-C ratio, the relationship between AIP and the outcomes are also influenced by TG levels. Recently, the concept of the “TG paradox” has been proposed by several studies, which found that TG levels are negatively associated with the risk of death in patients with CVD [47, 48]. The possible reason for this is that TG is significantly correlated with BMI [49], and patients with low TG levels have poor nutritional status and poor prognosis [50]. In our study, higher AIP predicted a higher risk of diabetes mortality, and lower AIP predicted a higher risk of non-diabetes mortality; this finding probably explains the U-shaped relationship of AIP with all-cause mortality. However, we did not explore the effect of hypertension drugs, which may affect lipid metabolism. We found that there was no significant correlation between AIP and all-cause mortality and CVD-specific mortality, probably because of the relatively small sample volume in our study. Thus, a cohort with more participants and longer follow-up is needed to validate our findings.

### Strengths and limitations

The strengths of our study include the use of a large national database and a prospective cohort study with a long follow-up period. In addition, NHANES uses standardized procedures for data collection, conducted by professional and trained personnel, including standard questionnaires, physical examinations, and laboratory tests. All blood samples were tested in the same cycle using standardized protocols, which greatly reduced potential bias. To our knowledge, this is the first study to explore the relationship between AIP and all-cause and specific mortality in the general population. In addition, we adjusted for covariates and performed subgroup analyses to explore these associations in different populations and non-linear association was observed.

However, some limitations of the study should be noted. First, the NHANES database uses death certificates and the level of accuracy in coding cases of death is susceptible to human reporting errors including, but not limited to, inaccurate cause of death assessments, compilation errors, and demographic classification errors. Second, we only included Americans, therefore, our findings may not be generalizable to other ethnic groups. Third, consecutive AIP changes during follow-up were not recorded. Fourth, although we have adjusted for several confounders, some potential confounding factors were not measured, such as dietary patterns and environmental factors. Despite these limitations, our results are still clinically important because we have demonstrated the association of AIP with the risk of all-cause and specific mortality.

### Perspectives and clinical applications

Our study suggests that monitoring AIP levels is an effective way to assess the risk of diabetes mortality. Keeping AIP at a certain level may have a positive effect on reducing all-cause mortality. While more research is needed to determine the mechanisms by which this effect exists, this study provides some insight into prevention strategies.

## Conclusion

In our study, there is a no significant association between high AIP levels and a high risk of all-cause and cardiovascular mortality, and we found the risk of diabetes mortality increased gradually with the increase of AIP, and the association was only found in women older than 65 years. Moreover, AIP was associated with all-cause mortality in a U-shape. This association could be explained by the finding that higher AIP predicted a higher risk of death from diabetes, and that lower AIP predicted a higher risk of death from non-diabetes causes. These findings suggest that controlling AIP levels may have a positive effect on reducing diabetes mortality.

### Electronic supplementary material

Supplementary Material 1

## Data Availability

Publicly available datasets were analyzed in this study. This data can be found here: https:// www. cdc. gov/ nchs/ nhanes/ index. htm.
