# Supplementary material for: Association between atherogenic index of plasma and all-cause mortality and specific-mortality: a nationwide population‑based cohort study
Source: Cardiovasc Diabetol. 2024 Jul 27;23:276. doi: 10.1186/s12933-024-02370-4 (PMC11283706; doi:10.1186/s12933-024-02370-4)
Supplement: Supplementary file 1 — Supplementary Material 1 [file 12933_2024_2370_MOESM1_ESM.docx]

**Supplementary File**

**Table 1S** Baseline characteristics of participants according to quintile categories

**Table 2S** Baseline characteristics of participants stratified by age group

**Table 3S** Baseline characteristics of participants according to age-adjusted proportions(95%CI)

**Table 4S** Association between AIP and all-cause mortality and specific-mortality excluding participants who died during the first two years of follow-up

**Table 5S** Association between AIP and all-cause mortality and specific-mortality excluding participants self-reported CVD at baseline

**Table 6S** Association between AIP and all-cause mortality and specific-mortality with an weighting procedure for the morning fasting subgroup

**Table 7S** Association between AIP and all-cause mortality and specific-mortality additionally adjusted for self-reported cancer status

**Table 1S** Baseline characteristics of participants according to quintile categories

|  | **Total** | Quintile 1 | Quintile 2 | Quintile 3 | Quintile 4 |
| --- | --- | --- | --- | --- | --- |
| **No. of participants** | 14063 | 3505 | 3552 | 3508 | 3498 |
| **Age (mean (SE))** | 46.64(0.29) | 44.26(0.48) | 45.87(0.44) | 48.02(0.41) | 48.02(0.40) |
| **Sex (%)** |  |  |  |  |  |
| Male | 6988,267031255.10 (49.0%) | 1275, 44992555.90 (33.7%) | 1692, 63989295.39 (45.8%) | 1808, 70137035.05 (51.8%) | 2213, 87912368.74 (64.4%) |
| **Race/Ethnics(%)** |  |  |  |  |  |
| Mexican American | 2440, 45706447.53 (8.4%) | 409,8078162.98  (6.0%) | 586, 11018066.25 (7.9%) | 663, 12408079.30 (9.2%) | 782, 14202139.01 (8.4%) |
| Non-Hispanic white | 6586, 383896661.6 (70.4%) | 1467, 89955875.63 (67.3%) | 1660, 99059017.97 (71.0%) | 1656, 95272602.91 (70.4%) | 1803, 99609165.08 (70.4%) |
| Non-Hispanic black | 2815, 55847231.01 (10.2%) | 1101, 21892453.62 (16.4%) | 752, 14801336.16 (10.6%) | 599, 11757661.72 (8.7%) | 363, 7395779.51 (10.2%) |
| **Current smoking (%)** | 3296, 133071265.6 (24.4%) | 654, 24602350.17 (18.4%) | 790, 32818322.25 (23.5%) | 851, 34207551.22 (25.3%) | 1001, 411443041.99 (30.3%) |
| **Current drinking (%)** | 8989, 384959429.1 (70.6%) | 2162, 93234737.16 (69.8%) | 2227, 97978302.93 (70.2%) | 2236, 94948484.48 (70.2%) | 2364, 98797904.52 (72.3%) |
| **Education level (%)** |  |  |  |  |  |
| Less than 9th grade | 1519, 31864201.03 (5.8%) | 211, 4619158.72  (3.5%) | 365, 7779967.05  (5.6%) | 411, 8694411.549 (6.4%) | 532, 10770663.71 (7.9%) |
| 9-11th grade | 2097, 62901121.57 (11.5%) | 459, 12516433.59  (9.4%) | 480, 14486570.84  (10.4%) | 552, 17574241.67  (13.0%) | 606, 18323875.48 (13.4%) |
| High school | 3556, 131005326.2 (24.0%) | 830, 27479807.53  (20.6%) | 933,33918862.19  (24.3%) | 915, 33850864.48 (25.0%) | 878, 35755791.95  (26.2%) |
| College | 3952, 168543830.8 (30.9%) | 1083, 42198402.98  (31.6%) | 987,43211593.81  (31.0%) | 939, 41008826.90 (30.3%) | 943, 42125007.09  (30.8%) |
| College graduate or above | 2925, 150538469.6 (27.6%) | 919,46733738.85  (35.0%) | 786,40176011.75  (28.8%) | 686, 34057587.31 (25.2%) | 534, 29571131.70 (21.6%) |
| **Annual household income (%)** |  |  |  |  |  |
| Under $20,000 | 3031, 77881907.08 (14.3%) | 686, 16748190.25 (12.5%) | 736, 19020144.40  (13.6%) | 758, 20344214.12 (15.0%) | 851, 21769358.31 (15.9%) |
| $20,000 to $45,000 | 4644, 154610812.9 (28.4%) | 1074, 34247710.52 (25.6%) | 1163, 38452855.34  (27.5%) | 1196, 40694663.62 (30.1%) | 1211, 41215583.40  (30.2%) |
| $45,000 to $75,000 | 2611, 119775139.9 (22.0%) | 673, 29052638.48 (21.7%) | 667, 31179875.30  (22.3%) | 634, 28235836.84  (20.9%) | 637, 31306789.32  (22.9%) |
| $75,000 to $100,000 | 1718, 94662986.08 (17.4%) | 436, 22718628.99 (17.0%) | 442, 24201397.41  (17.3%) | 458, 25059111.49  (18.5%) | 382, 22683848.20  (16.6%) |
| Over $100,000 | 1440, 80542142.00 (14.8%) | 487, 26399627.12 (19.8%) | 383, 22022961.58  (15.8%) | 315, 17173482.23  (12.7%) | 255, 14946071.07  (10.9%) |
| **BMI (mean (SE))** | 29.27(0.11) | 26.29(0.18) | 28.21(0.17) | 30.36(0.23) | 32.20(0.22) |
| **Exercised regularly (%)** | 6713, 231234956.0 (42.4%) | 1393, 47180415.27 (35.3%) | 1637, 55893609.62 (40.0%) | 1749, 59541441.04 (44.0%） | 1934, 68619490.10 (50.2%) |
| **Ever had CVD (%)** | 1501, 47991950.19 (8.8%) | 232, 7507541.72 (5.6%) | 336, 10206354.79 (7.3%) | 423, 13434343.83 (9.9%) | 510, 16843709.85 (12.3%) |
| **Ever had diabetes (%)** | 2278, 67279390.88 (12.3%) | 268, 7849702.356  (5.9%) | 444, 12277465.74 (8.8%) | 633, 18082968.56 (13.4%) | 933, 29069254.22 (21.3%) |
| **Ever had hypertension (%)** | 5676, 99897161.30  (36.7%) | 1041, 34625279.02 (25.9%) | 1328, 45977717.37 (32.9%) | 1571, 54028374.49 (39.9%) | 1736, 65265790.45 (47.8%) |
| **Ever had high cholesterol (%)** | 6720,261360137.80 (47.9%) | 705, 27330016.31 (20.5%) | 1183, 45460241.22 (32.6%) | 1728, 66763965.87 (49.3%) | 3104, 121805914.4 (89.2%) |

Continuous variables were presented as weighted mean (±SE), and categorical variables were presented as unweighted frequency, weighted frequency of participants (weighted percentage).

**Table 2S** Baseline characteristics of participants according to quintile categories stratified by age group

|  | **Total** | **< 65 year-old** | **≥ 65 year-old** |
| --- | --- | --- | --- |
| **No. of participants** | 14063 | 10978 | 3085 |
| **Age (mean (SE))** | 46.64(0.29) | 41.48(0.23) | 73.89(0.13) |
| **Sex (%)** |  |  |  |
| Male | 6988, 267031255.10 (49.0%) | 5455, 228115326  (49.8%) | 1533, 38915929.12  (44.8%) |
| **Race/Ethnics(%)** |  |  |  |
| Mexican American | 2440, 45706447.53 (8.4%) | 2082, 42706558.92  (9.3%) | 358, 2999888.61  (3.5%) |
| Non-Hispanic white | 6586, 383896661.6 (70.4%) | 4631, 311367522.4  (67.9%) | 1955, 72529139.15  (83.5%) |
| Non-Hispanic black | 2815, 55847231.01 (10.2%) | 2369, 50005571.85  (10.9%) | 446, 5841659.15  (6.7%) |
| **Current smoking (%)** | 3296, 133071265.6 (24.4%) | 2972, 124519902.5  (27.2%) | 324, 8551363.12  (9.8%) |
| **Current drinking (%)** | 8989, 384959429.1 (70.6%) | 7142, 331747153.7  (72.4%) | 1847, 53212275.42  (61.2%) |
| **Education level (%)** |  |  |  |
| Less than 9th grade | 1519, 31864201.03 (5.8%) | 908, 21446246.80  (4.7%) | 611, 10417954.23  (12.0%) |
| 9-11th grade | 2097, 62901121.57 (11.5%) | 1617, 51279826.68  (11.2%) | 480, 11621294.90  (13.4%) |
| High school | 3556, 131005326.2 (24.0%) | 2790, 107893004.4  (23.5%) | 766, 23112321.79  (26.6%) |
| College | 3952, 168543830.8 (30.9%) | 3278, 146226063.9  (31.9%) | 674, 22317766.90  (25.7%) |
| College graduate or above | 2925, 150538469.6 (27.6%) | 2379, 131187531.1  (28.6%) | 546, 19350938.46  (22.3%) |
| **Annual household income (%)** |  |  |  |
| Under $20,000 | 3031, 77881907.08 (14.3%) | 2131, 59237594.23  (12.9%) | 900, 18644312.86  (21.5%) |
| $20,000 to $45,000 | 4644, 154610812.9 (28.4%) | 3478, 120542319.0  (26.3%) | 1166, 34068493.94  (39.2%) |
| $45,000 to $75,000 | 2611, 119775139.9 (22.0%) | 2108, 103028862.5  (22.5%) | 503, 16746277.44  (19.3%) |
| $75,000 to $100,000 | 1718, 94662986.08 (17.4%) | 1499, 86994556.02  (19.0%) | 219, 7668430.06  (8.8%) |
| Over $100,000 | 1440, 80542142.00 (14.8%) | 1276, 73728469.06  (16.1%) | 164, 6813672.93  (7.8%) |
| **BMI (mean (SE))** | 29.27(0.11) | 29.21(0.13) | 29.59(0.23) |
| **Exercised regularly (%)** | 6713, 231234956.0 (42.4%) | 4827, 181816717.6  (39.7%) | 1886, 49418238.41  (56.9%) |
| **Ever had CVD (%)** | 1501, 47991950.19 (8.8%) | 595, 21970532.28  (4.8%) | 906, 26021417.91  (29.9%) |
| **Ever had diabetes (%)** | 2278, 67279390.88 (12.3%) | 1317, 43779386.41  (9.6%) | 961, 23500004.46  (27.0%) |
| **Ever had hypertension (%)** | 5676, 99897161.30  (36.7%) | 3401, 138234075.8  (30.2%) | 2275, 61663085.55  (71.0%) |
| **Ever had high cholesterol (%)** | 6720, 261360137.80 (47.9%) | 4738, 203488758.6  (44.4%) | 1982, 57871379.19  (66.6%) |
| **AIP (mean (SE))** | -0.03(0.01) | -0.03(0.01) | -0.02(0.01) |

Continuous variables were presented as weighted mean (±SE), and categorical variables were presented as unweighted frequency, weighted frequency of participants (weighted percentage).

**Table 3S** Baseline characteristics of participants according to age-adjusted proportions(95%CI)

|  | **Total** | **< 65 year-old** | **≥ 65 year-old** |
| --- | --- | --- | --- |
| **No. of participants** | 14063 | 10978 | 3085 |
| **Age(years)** | 46.64 (46.06-47.23) | 41.48(41.02-41.93) | 73.89(73.63-74.15) |
| **Sex (%)** |  |  |  |
| Male | 49.0 (48.1-49.9) | 49.8 (48.7-50.8) | 44.8 (43.2-46.4) |
| **Race/Ethnics(%)** |  |  |  |
| Mexican American | 8.4 (7.1-9.9) | 9.3 (8.0-10.9) | 3.5 (2.5-4.7) |
| Non-Hispanic white | 70.4 (67.7-73.0) | 67.9 (65.1-70.7) | 83.5 (81.0-85.6) |
| Non-Hispanic black | 10.2 (9.0-11.7) | 10.9 (9.6-12.4) | 6.7 (5.6-8.0) |
| **Current smoking (%)** | 24.4 (23.1-25.8) | 27.2 (25.7-28.7) | 9.8 (8.5-11.4) |
| **Current drinking (%)** | 70.6 (69.0-72.2) | 72.4 (70.7-74.0) | 61.2 (58.4-64.0) |
| **Education level (%)** |  |  |  |
| Less than 9th grade | 5.8 (5.3-6.5) | 4.7 (4.2-5.2) | 12.0 (10.4-13.7) |
| 9-11th grade | 11.5 (10.5-12.6) | 11.2 (10.2-12.3) | 13.4 (11.6-15.3) |
| High school | 24.0 (22.8-25.4) | 23.5 (22.2-25.0) | 26.6 (24.5-28.8) |
| College | 30.9 (29.7-32.1) | 31.9 (30.6-33.2) | 25.7 (23.7-27.8) |
| College graduate or above | 27.6 (25.8-29.5) | 28.6 (26.7-30.6) | 22.3 (19.6-25.1) |
| **Annual household income (%)** |  |  |  |
| Under $20,000 | 14.3 (13.1-15.6) | 12.9 (11.7-14.3) | 21.5 (19.3-23.8) |
| $20,000 to $45,000 | 28.4 (26.9-29.9) | 26.3 (24.7-27.9) | 39.2 (36.4-42.1) |
| $45,000 to $75,000 | 22.0 (20.6-23.5) | 22.5 (21.0-24.1) | 19.3 (16.8-22.0) |
| $75,000 to $100,000 | 17.4 (16.1-18.7) | 19.0 (17.6-20.5) | 8.8 (7.3-10.6) |
| Over $100,000 | 14.8 (13.0-16.7) | 16.1 (14.1-18.3) | 7.8 (6.2-9.9) |
| **BMI (kg/m^2^)** | 29.27(29.5-29.50) | 29.21(28.95-29.47) | 29.59(29.12-30.06) |
| **Exercised regularly (%)** | 42.4 (40.7-44.2) | 39.7 (37.8-41.5) | 56.9 (54.2-59.5) |
| **Ever had CVD (%)** | 8.8 (8.1-9.6) | 4.8 (4.3-5.4) | 29.9 (27.7-32.3) |
| **Ever had diabetes (%)** | 12.3 (11.6-13.2) | 9.6 (8.8-10.4) | 27.0 (25.2-29.0) |
| **Ever had hypertension (%)** | 36.7 (35.3-38.1) | 30.2 (28.9-31.5) | 71.0 (68.5-73.3) |
| **Ever had high cholesterol (%)** | 47.9 (46.7-49.2) | 44.4 (43.0-45.8) | 66.6 (64.4-68.7) |
| **AIP** | -0.03(-0.04 - -0.02) | -0.03(-0.04 - -0.02) | -0.02(-0.04 – 0.00) |

Continuous variables were presented as weighted mean (95%CI), and categorical variables were presented as weighted percentage(95%CI).

**Table 4S** Association between AIP and all-cause mortality and specific-mortality excluding participants who died during the first two years of follow-up

|  |  | **Model 1** | | **Model 2** | | **Model 3** | |  |
| --- | --- | --- | --- | --- | --- | --- | --- | --- |
|  | Events/total | HR (95%CI)^a^ | *p* | HR (95%CI)^a^ | *p* | HR (95%CI)^a^ | *p* |  |
| **All-cause mortality** |  |  |  |  |  |  |  |  |
| Quartile 1 | 373/3505 | reference | | reference | | reference | |  |
| Quartile 2 | 508/3552 | 1.26(1.02,0.56) | 0.030 | 1.02(0.82,1.27) | 0.849 | 0.92(0.74,0.15) | 0.462 |  |
| Quartile 3 | 583/3508 | 1.51(1.21,1.87) | 0.000 | 1.06(0.85,1.31) | 0.622 | 0.93(0.75,0.15) | 0.484 |  |
| Quartile 4 | 613/3498 | 1.66(1.38,2.02) | 0.000 | 1.23(1.02,1.47) | 0.028 | 1.03(0.86,1.23) | 0.762 |  |
| **Diabetes mortality** |  |  |  |  |  |  |  |  |
| Quartile 1 | 27/3505 | reference | | reference | | reference | |  |
| Quartile 2 | 41/3552 | 2.02(0.95,4.30) | 0.069 | 1.65(0.77,3.56) | 0.195 | 1.35(0.64,2.85) | 0.431 |  |
| Quartile 3 | 65/3508 | 2.66(1.22,5.80) | 0.015 | 1.89(0.87,4.14) | 0.108 | 1.44(0.68,3.07) | 0.343 |  |
| Quartile 4 | 129/3498 | 6.35(3.14,12.84) | 0.000 | 4.54(2.21,9.35) | 0.000 | 2.96(1.45,6.08) | 0.003 |  |
| **CVD mortality** |  |  |  |  |  |  |  |  |
| Quartile 1 | 148/3505 | reference | | reference | | reference | |  |
| Quartile 2 | 200/3552 | 1.43(1.03,1.97) | 0.031 | 1.13(0.82,1.56) | 0.435 | 1.01(0.74,1.37) | 0.957 |  |
| Quartile 3 | 248/3508 | 1.59(1.18,2.14) | 0.003 | 1.10(0.80,1.52) | 0.565 | 0.94(0.69,1.28) | 0.667 |  |
| Quartile 4 | 258/3498 | 1.79(1.34,2.39) | 0.000 | 1.34(1.00,1.80) | 0.053 | 1.05(0.77,1.42) | 0.760 |  |
| **Cancer mortality** | |  |  |  |  |  |  |  |
| Quartile 1 | | 80/3505 | reference | | reference | | reference | |
| Quartile 2 | | 126/3552 | 1.56(0.95,2.56) | 0.080 | 1.27(0.77,2.09) | 0.345 | 1.23(0.73,2.05) | 0.435 |
| Quartile 3 | | 131/3508 | 1.50(0.94,2.38) | 0.088 | 1.06(0.67,1.67) | 0.812 | 1.02(0.63,1.63) | 0.941 |
| Quartile 4 | | 139/3498 | 1.94(1.25,3.01) | 0.004 | 1.41(0.92,2.16) | 0.112 | 1.36(0.85,2.17) | 0.202 |

Model 1, unadjusted;

Model 2, adjusted for age and sex;

Model 3, adjusted for age, sex, race/ethnicity, education level, annual household income, body mass index, current smoking, current alcohol drinking, moderate to vigorous activity regularly, self-reported of CVD, diabetes, and high cholesterol.

**Table 5S** Association between AIP and all-cause mortality and specific-mortality excluding participants CVD diagnosed at baseline

|  |  | **Model 1** | | **Model 2** | | **Model 3** | |  |
| --- | --- | --- | --- | --- | --- | --- | --- | --- |
|  | Events/total | HR (95%CI)^a^ | *p* | HR (95%CI)^a^ | *p* | HR (95%CI)^a^ | *p* |  |
| **All-cause mortality** |  |  |  |  |  |  |  |  |
| Quartile 1 | 244/3130 | reference | | reference | | reference | |  |
| Quartile 2 | 342/3168 | 1.28(1.02,1.61) | 0.030 | 1.05(0.84,1.32) | 0.643 | 0.98(0.78,1.22) | 0.800 |  |
| Quartile 3 | 380/3134 | 1.50(1.20,1.89) | 0.001 | 1.14(0.91,1.43) | 0.253 | 1.02(0.82,1.28) | 0.831 |  |
| Quartile 4 | 389/3130 | 1.50(1.21,1.86) | 0.000 | 1.19(0.96,1.47) | 0.109 | 1.03(0.84,1.26) | 0.830 |  |
| **Diabetes mortality** |  |  |  |  |  |  |  |  |
| Quartile 1 | 17/3130 | reference | | reference | | reference | |  |
| Quartile 2 | 24/3168 | 1.26(0.52,3.07) | 0.602 | 1.03(0.42,2.52) | 0.947 | 0.94(0.39,2.24) | 0.879 |  |
| Quartile 3 | 41/3134 | 1.96(0.90,4.23) | 0.087 | 1.46(0.66,3.24) | 0.347 | 1.29(0.58,2.90) | 0.531 |  |
| Quartile 4 | 71/3130 | 3.79(1.71,8.39) | 0.001 | 2.84(1.25,6.44) | 0.013 | 2.37(1.04,5.38) | 0.040 |  |
| **CVD mortality** |  |  |  |  |  |  |  |  |
| Quartile 1 | 85/3130 | reference | | reference | | reference | |  |
| Quartile 2 | 124/3168 | 1.47(1.02,2.12) | 0.038 | 1.16(0.81,1.66) | 0.414 | 1.07(0.75,1.53) | 0.714 |  |
| Quartile 3 | 143/3134 | 1.66(1.17,2.34) | 0.005 | 1.22(0.84,1.78) | 0.299 | 1.08(0.74,1.58) | 0.675 |  |
| Quartile 4 | 145/3130 | 1.58(1.08,2.31) | 0.018 | 1.28(0.88,1.87) | 0.201 | 1.08(0.73,1.58) | 0.707 |  |
| **Cancer mortality** | |  |  |  |  |  |  |  |
| Quartile 1 | | 61/3130 | reference | | reference | | reference | |
| Quartile 2 | | 89/3168 | 1.59(1.04,2.43) | 0.033 | 1.33(0.87,2.04) | 0.185 | 1.29(0.84,1.98) | 0.243 |
| Quartile 3 | | 98/3134 | 1.67(1.11,2.50) | 0.015 | 1.29(0.87,1.92) | 0.208 | 1.23(0.82,1.85) | 0.306 |
| Quartile 4 | | 96/3130 | 1.85(1.24,2.75) | 0.003 | 1.50(1.01,2.22) | 0.043 | 1.42(0.96,2.10) | 0.080 |

Model 1, unadjusted;

Model 2, adjusted for age and sex;

Model 3, adjusted for age, sex, race/ethnicity, education level, annual household income, body mass index, current smoking, current alcohol drinking, moderate to vigorous activity regularly, self-reported of diabetes and high cholesterol.

**Table 6S** Association between AIP and all-cause mortality and specific-mortality with an weighting procedure for the morning fasting subgroup

|  |  | **Model 1** | | **Model 2** | | **Model 3** | |  |
| --- | --- | --- | --- | --- | --- | --- | --- | --- |
|  | Events/total | HR (95%CI)^a^ | *p* | HR (95%CI)^a^ | *p* | HR (95%CI)^a^ | *p* |  |
| **All-cause mortality** |  |  |  |  |  |  |  |  |
| Quartile 1 | 295/2809 | reference | | reference | | reference | |  |
| Quartile 2 | 366/2812 | 1.12(0.92,1.36) | 0.258 | 0.89(0.73,1.07) | 0.201 | 0.81(0.67,0.99) | 0.034 |  |
| Quartile 3 | 440/2844 | 1.26(1.01,1.56) | 0.037 | 0.97(0.78,1.21) | 0.794 | 0.85(0.68,1.06) | 0.139 |  |
| Quartile 4 | 467/2803 | 1.59(1.31,1.94) | 0.000 | 1.06(0.86,1.31) | 0.583 | 0.90(0.73,1.10) | 0.286 |  |
| Quartile 5 | 509/2795 | 1.66(1.36,2.04) | 0.000 | 1.22(1.01,1.48) | 0.040 | 1.00(0.83,1.21) | 0.987 |  |
| **Diabetes mortality** |  |  |  |  |  |  |  |  |
| Quartile 1 | 21/2809 | reference | | reference | | reference | |  |
| Quartile 2 | 31/2812 | 1.53(0.79,2.97) | 0.209 | 1.22(0.63,2.36) | 0.550 | 1.00(0.51,1.97) | 0.994 |  |
| Quartile 3 | 42/2844 | 1.70(0.86,3.35) | 0.129 | 1.30(0.66,2.58) | 0.449 | 0.98(0.50,1.95) | 0.958 |  |
| Quartile 4 | 55/2803 | 2.60(1.35,4.98) | 0.005 | 1.71(0.88,3.32) | 0.111 | 1.17(0.61,2.24) | 0.645 |  |
| Quartile 5 | 113/2795 | 4.76(2.57,8.82) | 0.000 | 3.33(1.79,6.21) | 0.000 | 2.07(1.09,3.94) | 0.028 |  |
| **CVD mortality** |  |  |  |  |  |  |  |  |
| Quartile 1 | 119/2809 | reference | | reference | | reference | |  |
| Quartile 2 | 144/2812 | 1.20(0.88,1.63) | 0.256 | 0.92(0.69,1.23) | 0.575 | 0.81(0.61,1.08) | 0.145 |  |
| Quartile 3 | 181/2844 | 1.39(1.03,1.87) | 0.029 | 1.06(0.79,1.43) | 0.687 | 0.89(0.66,1.19) | 0.421 |  |
| Quartile 4 | 199/2803 | 1.81(1.33,2.48) | 0.000 | 1.20(0.86,1.66) | 0.276 | 0.94(0.68,1.30) | 0.708 |  |
| Quartile 5 | 211/2795 | 1.82(1.35,2.45) | 0.000 | 1.36(1.02,1.81) | 0.037 | 1.02(0.76,1.37) | 0.896 |  |
| **Cancer mortality** | |  |  |  |  |  |  |  |
| Quartile 1 | | 62/2809 | reference | | reference | | reference | |
| Quartile 2 | | 90/2812 | 1.40(0.91,2.17) | 0.126 | 1.12(0.73,1.73) | 0.590 | 1.12(0.72,1.74) | 0.625 |
| Quartile 3 | | 98/2844 | 1.26(0.81,1.98) | 0.303 | 0.98(0.62,1.53) | 0.915 | 0.95(0.59,1.53) | 0.828 |
| Quartile 4 | | 111/2803 | 1.60(1.01,2.52) | 0.044 | 1.07(0.68,1.68) | 0.773 | 1.05(0.65,1.69) | 0.847 |
| Quartile 5 | | 115/2795 | 2.01(1.29,3.13) | 0.002 | 1.46(0.95,2.24) | 0.084 | 1.43(0.90,2.29) | 0.129 |

Model 1, unadjusted;

Model 2, adjusted for age and sex;

Model 3, adjusted for age, sex, race/ethnicity, education level, annual household income, body mass index, current smoking, current alcohol drinking, moderate to vigorous activity regularly, self-reported of CVD, diabetes, hypertension, and high cholesterol.

**Table 7S** Association between AIP and all-cause mortality and specific-mortality additionally adjusted for self-reported cancer status

|  |  | **Model 1** | | **Model 2** | | **Model 3** | |  |
| --- | --- | --- | --- | --- | --- | --- | --- | --- |
|  | Events/total | HR (95%CI)^a^ | *p* | HR (95%CI)^a^ | *p* | HR (95%CI)^a^ | *p* |  |
| **All-cause mortality** |  |  |  |  |  |  |  |  |
| Quartile 1 | 373/3505 | reference | | reference | | reference | |  |
| Quartile 2 | 508/3552 | 1.22(1.03,1.46) | 0.023 | 1.01(0.84,1.21) | 0.921 | 0.93(0.78,1.12) | 0.449 |  |
| Quartile 3 | 583/3508 | 1.50(1.27,1.78) | 0.000 | 1.09(0.92,1.30) | 0.329 | 0.96(0.81,1.14) | 0.658 |  |
| Quartile 4 | 613/3498 | 1.62(1.37,1.91) | 0.000 | 1.24(1.05,1.45) | 0.010 | 1.04(0.89,1.22) | 0.595 |  |
| **Diabetes mortality** |  |  |  |  |  |  |  |  |
| Quartile 1 | 27/3505 | reference | | reference | | reference | |  |
| Quartile 2 | 41/3552 | 1.39(0.74,2.62) | 0.306 | 1.16(0.60,2.23) | 0.661 | 0.96(0.49,1.86) | 0.891 |  |
| Quartile 3 | 65/3508 | 2.04(1.15,3.64) | 0.016 | 1.50(0.84,2.67) | 0.168 | 1.14(0.64,2.02) | 0.649 |  |
| Quartile 4 | 129/3498 | 4.40(2.53,7.65) | 0.000 | 3.30(1.88,5.81) | 0.000 | 2.16(1.20,3.89) | 0.011 |  |
| **CVD mortality** |  |  |  |  |  |  |  |  |
| Quartile 1 | 148/3505 | reference | | reference | | reference | |  |
| Quartile 2 | 200/3552 | 1.34(1.01,1.77) | 0.042 | 1.09(0.82,1.44) | 0.549 | 0.98(0.75,1.28) | 0.888 |  |
| Quartile 3 | 248/3508 | 1.62(1.27,2.06) | 0.000 | 1.17(0.89,1.52) | 0.256 | 0.98(0.76,1.27) | 0.882 |  |
| Quartile 4 | 258/3498 | 1.81(1.40,2.35) | 0.000 | 1.41(1.09,1.84) | 0.011 | 1.10(0.83,1.44) | 0.511 |  |
| **Cancer mortality** | |  |  |  |  |  |  |  |
| Quartile 1 | | 80/3505 | reference | | reference | | reference | |
| Quartile 2 | | 126/3552 | 1.50(0.97,2.32) | 0.067 | 1.24(0.81,1.91) | 0.325 | 1.22(0.79,1.91) | 0.369 |
| Quartile 3 | | 131/3508 | 1.59(1.08,2.35) | 0.020 | 1.16(0.79,1.69) | 0.446 | 1.14(0.77,1.70) | 0.510 |
| Quartile 4 | | 139/3498 | 1.85(1.24,2.76) | 0.003 | 1.40(0.94,2.07) | 0.093 | 1.40(0.92,2.13) | 0.120 |

Model 1, unadjusted;

Model 2, adjusted for age and sex;

Model 3, adjusted for age, sex, race/ethnicity, education level, annual household income, body mass index, current smoking, current alcohol drinking, moderate to vigorous activity regularly, self-reported of CVD, diabetes, hypertension, high cholesterol and cancer.
